# Impact of baseline beta-blocker use on inotrope response and clinical outcomes in cardiogenic shock: a subgroup analysis of the DOREMI trial

**DOI:** 10.1186/s13054-021-03706-2

**Published:** 2021-08-10

**Authors:** Pietro Di Santo, Rebecca Mathew, Richard G. Jung, Trevor Simard, Stephanie Skanes, Brennan Mao, F. Daniel Ramirez, Jeffrey A. Marbach, Omar Abdel-Razek, Pouya Motazedian, Simon Parlow, Kevin E. Boczar, Gianni D’Egidio, Steven Hawken, Jordan Bernick, George A. Wells, Alexander Dick, Derek Y. So, Christopher Glover, Juan J. Russo, Caroline McGuinty, Benjamin Hibbert, the CAPITAL DOREMI investigators

**Affiliations:** 1 CAPITAL Research Group, Division of Cardiology, University of Ottawa Heart Institute, 40 Ruskin Street, H-4238, Ottawa, ON K1Y 4W7 Canada; 2 Faculty of Medicine, University of Ottawa, Ottawa, ON Canada; 3 School of Epidemiology and Public Health, University of Ottawa, Ottawa, ON Canada; 4 Division of Critical Care, Department of Medicine, University of Ottawa, Ottawa, ON Canada; 5 Department of Cellular and Molecular Medicine, University of Ottawa, Ottawa, ON Canada; 6 Department of Cardiovascular Medicine, Mayo Clinic, Rochester, MN USA; 7 Hôpital Cardiologique du Haut Lévêque, CHU Bordeaux, Bordeaux-Pessac, France; 8 LIRYC (L’Institut de Rythmologie Et Modélisation Cardiaque), Bordeaux-Pessac, France; 9 Division of Critical Care, Tufts Medical Center, Boston, MA USA; 10 Department of Medicine, Cumming School of Medicine, Calgary, AB Canada; 11 Ottawa Hospital Research Institute, Ottawa, ON Canada; 12 Cardiovascular Research Methods Centre, University of Ottawa Heart Institute, Ottawa, ON Canada

**Keywords:** Cardiogenic shock, Beta-blocker, Inotropes, Milrinone, Dobutamine

## Abstract

**Background:**

Cardiogenic shock (CS) is associated with significant morbidity and mortality. The impact of beta-blocker (BB) use on patients who develop CS remains unknown. We sought to evaluate the clinical outcomes and hemodynamic response profiles in patients treated with BB in the 24 h prior to the development of CS.

**Methods:**

Patients with CS enrolled in the DObutamine compaREd to MIlrinone trial were analyzed. The primary outcome was a composite of all-cause mortality, resuscitated cardiac arrest, need for cardiac transplant or mechanical circulatory support, non-fatal myocardial infarction, transient ischemic attack or stroke, or initiation of renal replacement therapy. Secondary outcomes included the individual components of the primary composite and hemodynamic response profiles derived from pulmonary artery catheters.

**Results:**

Among 192 participants, 93 patients (48%) had received BB therapy. The primary outcome occurred in 47 patients (51%) in the BB group and in 52 (53%) in the no BB group (RR 0.96; 95% CI 0.73–1.27; *P* = 0.78) throughout the in-hospital period. There were fewer early deaths in the BB group (RR 0.41; 95% CI 0.18–0.95; *P* = 0.03). There were no differences in other individual components of the primary outcome or in hemodynamic response between the two groups throughout the remainder of the hospitalization.

**Conclusions:**

BB therapy in the 24 h preceding the development of CS did not negatively influence clinical outcomes or hemodynamic parameters. On the contrary, BB use was associated with fewer deaths in the early resuscitation period, suggesting a paradoxically protective effect in patients with CS.

*Trial registration* ClinicalTrials.gov Identifier: NCT03207165

## Background

Cardiogenic shock (CS) is defined as a state of low cardiac output resulting in clinical and biochemical manifestations of end-organ hypoperfusion.^1,2^ The evidence base to guide the medical management of patients with CS has been limited to date. In this context, the recent DObutamine compaREd to MIlrinone (DOREMI) trial compared dobutamine and milrinone in patients with CS, finding no differences in outcomes between inotropes.^3^

Beta-blockers (BBs) are commonly used in patients with cardiovascular disease, including for the management of heart failure with reduced ejection fraction^4−6^ and cardiac arrhythmias.^7,8^ Accordingly, it is not uncommon for patients presenting with CS to have recently received BB therapy—a treatment with known hemodynamic effects. The negative impact of inotropes with concomitant BB therapy in acute decompensated heart failure has been established previously.^9,10^ Moreover, dobutamine—a synthetic catecholamine with beta-1 and beta-2 receptor agonism—has been suggested to be less effective as an inotrope in the setting of BB therapy.^11^ The impact of baseline BB therapy on clinical outcomes or the efficacy of dobutamine in BB-treated patients presenting with CS has not been previously described.

We hypothesized that patients with BB use in the 24 h before developing CS would have worse clinical outcomes and hemodynamic parameters owing to its negative inotropic effects. We further hypothesized that dobutamine would be less effective as an inotrope in this context. Therefore, we evaluated the clinical outcomes and hemodynamic response profiles in patients with CS treated with BB as a subgroup analysis of the DOREMI trial.

## Methods

### Study design

This current study represents a post-hoc subgroup analysis of the DOREMI trial. The DOREMI protocol, eligibility criteria, and methods have been reported previously.^3^ In brief, the DOREMI trial was a randomized, double-blind clinical trial of dobutamine versus milrinone in patients with CS. Individuals who had CS requiring admission to a cardiac intensive care unit (CICU) were eligible. Patients were recruited from a single quaternary care cardiac institute between September 1, 2017 and May 17, 2020 in Ottawa, Canada. Eligible patients were aged 18 years or older and met the Society for Cardiovascular Angiography and Interventions (SCAI) definitions of CS stages B through E.^12^ Patients were excluded if they presented with an out-of-hospital cardiac arrest, were pregnant, had milrinone or dobutamine initiated prior to randomization, the treating physician was of the opinion that the patient was not eligible for the study, the patient was participating in another interventional trial, or if the patient or substitute decision maker was unable to provide written informed consent. Participants were randomized using a computer-generated random sequence to receive either milrinone or dobutamine in a 1:1 ratio. The treating physicians, patient, local investigators, and all research personnel were blinded to the treatment allocation. Following randomization, participants were started on either milrinone or dobutamine using a standardized scale, ranging from stage 1 to 5, which corresponded to 2.5, 5.0, 7.5, 10.0 and > 10.0 µg/kg/min for dobutamine and 0.125, 0.250, 0.375, 0.500 and > 0.500 µg/kg/min for milrinone. Following the initiation of inotrope therapy, a member of the primary treating team reassessed the participant at pre-specified intervals (4, 8, 12, 18, 24, 36, 48, 60, 72 h then daily thereafter). At each reassessment, the treating team made a decision regarding the inotrope dose (maintain, increase, or decrease) based on clinical judgment. Pulmonary artery catheters were not routinely used in managing patients with CS but were permitted at the discretion of the treating team. Clinical and biochemical information were recorded at inotrope initiation and at each reassessment. Written informed consent was obtained from all participants or their substitute decision maker. Ethics approval was obtained from the Ottawa Health Science Network Research Ethics Board and the study was conducted in accordance with the Helsinki Declaration.

### Study end points

The primary end point of this subgroup analysis was the composite of all-cause mortality, resuscitated cardiac arrest, need for cardiac transplantation or mechanical circulatory support, non-fatal myocardial infarction (MI), transient ischemic attack or stroke, or initiation of renal replacement therapy (RRT). This was evaluated in both the early treatment period (within 48 h of inotropic therapy initiation) and over the entire in-hospital period. The early treatment period was deemed clinically relevant as it was reasoned that the BB effect would be negligible beyond this period. Secondary efficacy end points included each individual component of the primary composite outcome and changes in hemodynamic parameters, including cardiac index, systemic vascular resistance, and pulmonary capillary wedge pressure. To account for the competing risk of death, resuscitated cardiac arrest was considered as ‘aborted death’ and the analysis performed combining resuscitated arrest and all-cause mortality. Secondary safety end points were CICU length of stay ≥ 7 days, acute kidney injury, arrhythmia requiring intravenous anti-arrhythmic therapy or electrical cardioversion, atrial arrhythmias requiring medical intervention, ventricular arrhythmias, need for initiation or increase of intravenous or oral anti-arrhythmic therapy, and need for initiation or increase of vasopressor therapy.

Following completion of the trial, we performed post-hoc analyses of clinical and biochemical parameters, including heart rate, mean arterial pressure, hourly urine output, vasoactive-inotropic score,^13^ serum lactate, and creatinine among the BB and no BB groups. As part of this post-hoc analysis, we evaluated the primary composite outcome, heart rate, mean arterial pressure, and vasoactive-inotropic scores in (1) analyses restricted to patients who had received BB therapy stratified by inotrope type and (2) in analyses restricted to patients randomized to dobutamine.

### Statistical analysis

Data were analyzed according to the intention-to-treat principle. Categorical data are expressed as numbers and percentages and continuous variables are expressed as means (SDs) for normally distributed variables or otherwise as medians (IQRs). We analyzed the primary composite outcome and its individual components as relative risks using unadjusted and pre-specified adjusted Chi-square analyses. The covariates included in the adjusted model were age, sex, BB use, type of inotrope (i.e. dobutamine or milrinone), and history of atrial fibrillation. Survival curves were constructed using Kaplan–Meier estimates. Secondary outcomes were analyzed using Chi square or Fisher's exact tests, as appropriate. For variables measured more than once throughout the study, a repeated measure mixed model for continuous variables and a cumulative logistic regression model for ordinal variables were used to test the association between BB use and outcome. All reported *P *values are two-sided and a value less than 0.05 was considered statistically significant. Analyses were performed using SAS (version 9.4, SAS Institute, Cary, North Carolina, USA).

## Results

### Study population

**Fig. 1:**
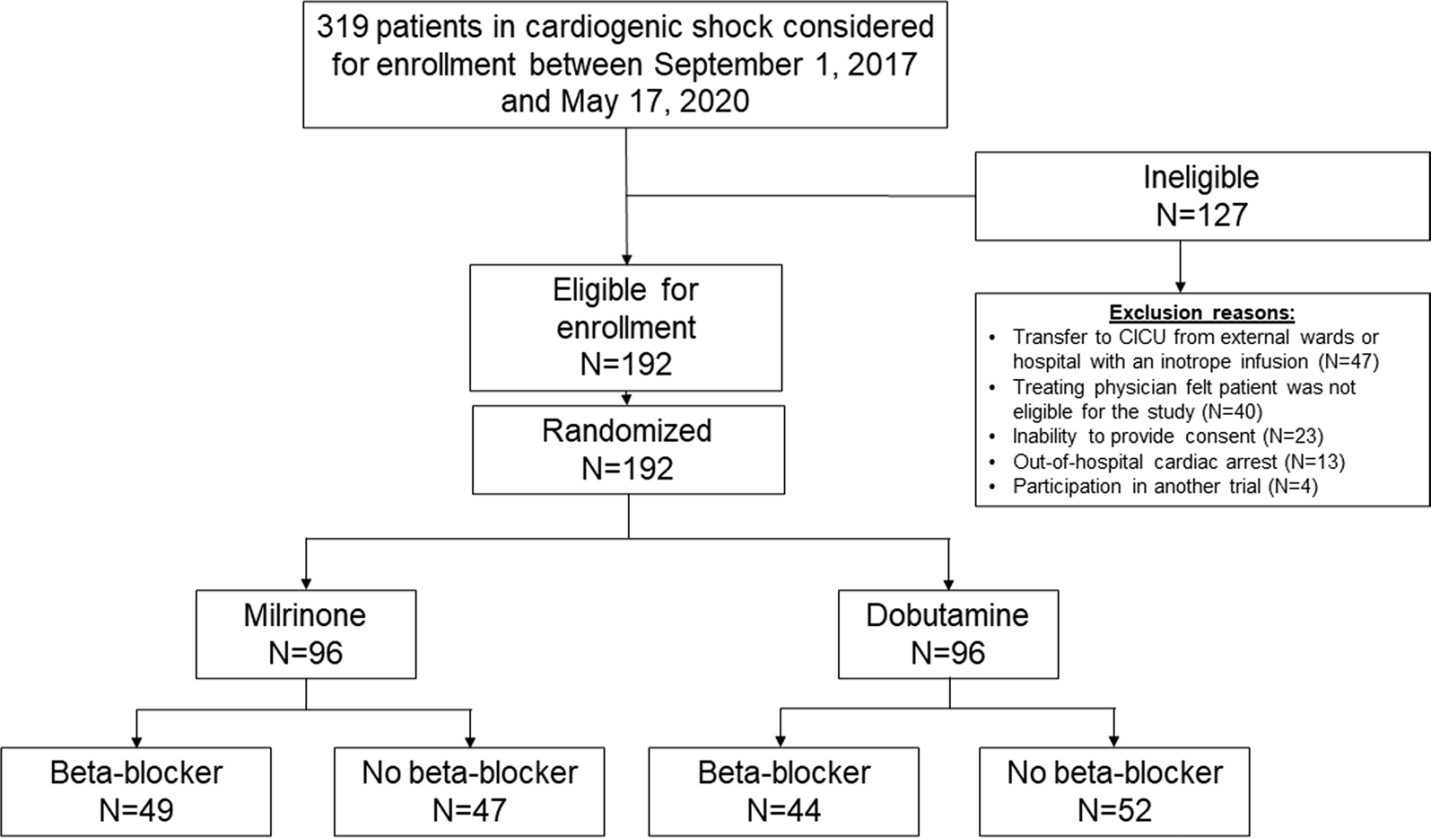
Study flow diagram. All participants in the DObutamine compaREd to MIlrinone study were included in the subgroup analysis. There were 49 (51%) patients on beta-blockers in the milrinone group and 44 (46%) patients on beta-blockers in the dobutamine group for a total of 93 (48%) patients on beta-blockers

All 192 participants in the DOREMI study were included in the analysis. There were 49 (51%) patients on BBs in the milrinone group and 44 (46%) patients on BBs in the dobutamine group for a total of 93 (48%) patients on BBs (Fig. 1).

Baseline characteristics are summarized in Table 1. A history of previous MI, previous percutaneous coronary intervention, previous coronary artery bypass graft surgery, and atrial fibrillation were more common in the BB group. Medical therapy including anticoagulation, angiotensin converting enzyme inhibitor, angiotensin-II receptor blocker, or angiotensin receptor neprilysin inhibitor, and mineralocorticoid receptor antagonist therapy were also more frequent in the BB group. At randomization, 10 participants had an intra-aortic balloon pump in place (3 in the BB group and 7 in the no BB group) and 23 participants had a pulmonary artery catheter (9 in BB group and 14 in the no BB group). The median serum lactate level was similarly elevated to 3.0 mmol/L (1.8–4.5 mmol/L) in the BB group and 2.8 mmol/L (1.8–4.4 mmol/L) in the no BB group.

**Table 1:** Baseline characteristics*

	Beta-blocker (n = 93)	No beta-blocker use (n = 99)
Age, years	70.7 ± 11.2	70.2 ± 14.0
Females—no. (%)	35 (38%)	35 (35%)
Body mass index, median (IQR)	26.3 (23.0–30.7)	26.1 (22.9–31.2)
*Ethnicity—no. (%)*		
Caucasian	81 (87%)	84 (85%)
Non-Caucasian	12 (13%)	15 (15%)
*Left ventricular function—no. (%)*		
Left ventricular ejection fraction, median (IQR)—%	25 (18–33)	28 (20–45)
Etiology of ventricular dysfunction		
Ischemic	58 (62%)	70 (71%)
Non-ischemic	35 (38%)	28 (29%)
*Co-morbidities—no. (%)*		
Previous myocardial infarction	40 (43%)	28 (28%)
Previous percutaneous coronary intervention	32 (34%)	17 (17%)
Previous coronary artery bypass grafting	27 (29%)	12 (12%)
Previous stroke/transient ischemic attack	16 (17%)	12 (12%)
Diabetes mellitus	48 (52%)	54 (55%)
Atrial fibrillation	62 (67%)	33 (33%)
Chronic kidney disease	28 (30%)	22 (22%)
Chronic liver disease	7 (8%)	6 (6%)
*Medications received in preceding 24 h prior to randomization—no. (%)*		
Aspirin	53 (57%)	74 (75%)
P2Y12 inhibitor	37 (40%)	62 (63%)
Warfarin	15 (16%)	6 (6%)
Direct oral anticoagulant	28 (30%)	11 (11%)
Statin	65 (70%)	61 (62%)
Angiotensin converting enzyme inhibitor, angiotensin-II receptor blocker, or angiotensin receptor neprolysin inhibitor	61 (66%)	24 (24%)
Mineralocorticoid receptor antagonist	26 (28%)	3 (3%)
Nitrates/hydralazine	13 (14%)	10 (10%)
Diuretic	77 (83%)	74 (75%)
Digoxin	11 (12%)	3 (3%)
Amiodarone	38 (41%)	29 (29%)
*Society for Cardiovascular Angiography and Interventions cardiogenic shock class—no. (%)*		
Class A	0 (0%)	0 (0%)
Class B	6 (6%)	5 (5%)
Class C	78 (84%)	77 (78%)
Class D	6 (16%)	16 (16%)
Class E	3 (2%)	1 (1%)
*Clinical parameters at initiation of inotropes*		
Heart rate, median (IQR), beats per minute—no. (%)	90 (72–105)	92 (76–105)
Mean arterial pressure, median (IQR), mmHg	78 (69–85)	72 (66–83)
No. of patients on vasopressors—no. (%)	38 (41%)	54 (55%)
Intra-aortic balloon pump—no. (%)	3 (3%)	7 (7%)
Vasoactive-inotropic score, median (IQR)	2.5 (1.3–11.3)	4.4 (1.3–15.3)
Pulmonary artery catheter in-situ—no. (%)	9 (10%)	14 (14%)
Cardiac index, median (IQR), liters/min/metres^2^	1.8 (1.7–2.0)	1.6 (1.3–1.8)
Systemic vascular resistance, median (IQR), dynes⋅sec⋅cm^−5^	1872 (1443–2082)	1505 (1417–2052)
Mixed venous oxygen saturation, median (IQR), %	62 (28–73)	56 (54–65)
No. of participants requiring non-invasive ventilation	9 (10%)	8 (8%)
No. of participants requiring invasive ventilation	14 (15%)	26 (26%)
*Laboratory values at initiation of inotropes*		
Hemoglobin, median (IQR), g/L	113 (99–134)	118 (103–135)
Creatinine, median (IQR), µmol/L	161 (127–234)	141 (110–201)
Lactate, median (IQR), mmol/L	3.0 (1.8–4.5)	2.8 (1.8–4.4)
Aspartate transaminase, median (IQR), units/L	111 (41–342)	200 (64–559)

Plus minus values are mean ± standard deviation. IQR denotes interquartile range

Within the BB group, 41 (44%) patients were taking metoprolol at a median dose of 37.5 mg twice daily (IQR 25–50 mg twice daily), 42 (45%) were taking bisoprolol at a median dose of 5 mg daily (IQR 2.5–5 mg daily), and 6 (6%) were taking carvedilol at a median dose of 6.25 mg twice daily (IQR 6.25–12 mg twice daily). There were 3 (3%) patients taking atenolol at a median dose of 50 mg daily (IQR 50–50 mg daily).

### Primary outcome

**Fig. 2:**
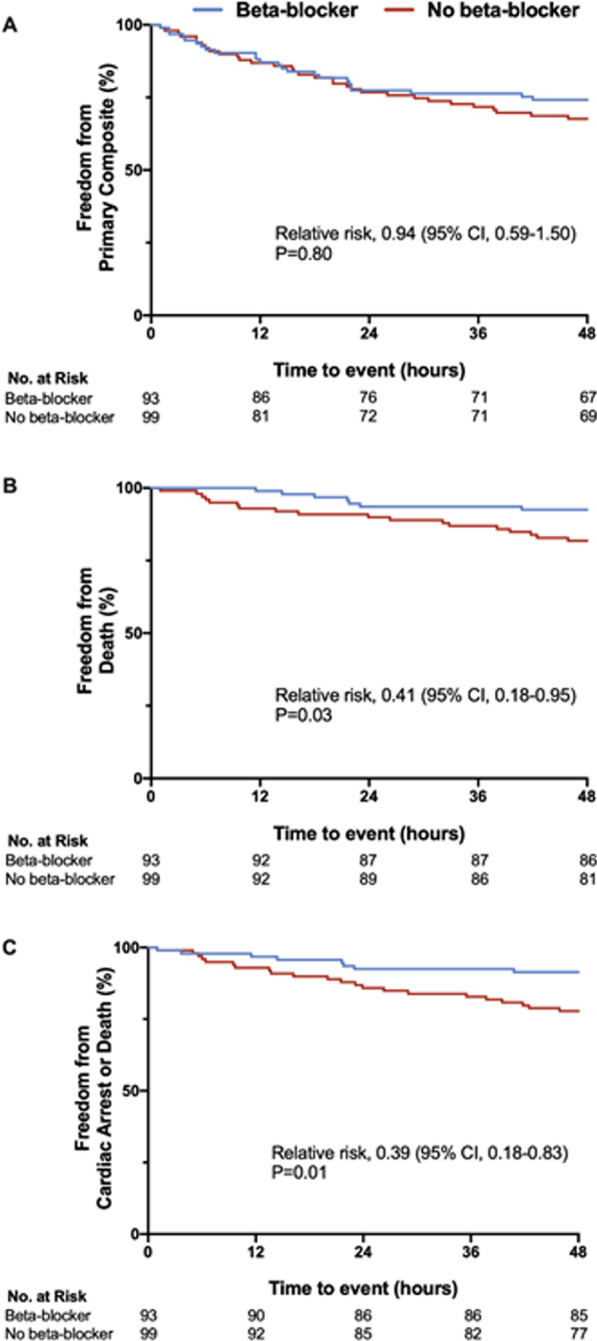
Time-to-event curves for the primary endpoint, death, and resuscitated cardiac arrest in the early resuscitation period. **a** Primary composite outcome, **b** all-cause mortality, and **c** death or resuscitated cardiac arrest within the first 48-h of initiation of inotropic therapy. Survival curves were constructed using Kaplan–Meier estimates. Adjusted relative risk using Chi-square testing was used for the primary outcome including age, sex, beta-blocker use, type of inotrope, and history of atrial fibrillation as covariates. Unadjusted relative risks using Chi-square testing was used for all-cause mortality and death or resuscitated cardiac arrest

The composite primary outcome occurred in 24 (26%) and 32 (32%) participants during the early period in the BB and no BB groups, respectively (adjusted relative risk (aRR) 0.94; 95% confidence interval (CI) 0.59–1.50; *P* = 0.80) (Table 2; Fig. 2a). There was also no difference in the primary composite outcome over the entire in-hospital period (aRR 1.14; 95% CI 0.85–1.53; *P* = 0.38).

**Table 2:** Primary composite outcome and individual subcomponents during the initial 48 h and total in-hospital length of stay

End point	Early outcomes (48 h)						In-hospital outcomes					
	Beta-blocker (n = 93)	No beta-blocker (n = 99)	Crude RR (95% CI)	*P* value	Adjusted RR^a^ (95% CI)	*P* value	Beta-blocker (n = 93)	No beta-blocker (n = 99)	Crude RR (95% CI)	*P* value	Adjusted RR^a^ (95% CI)	*P* value
Primary composite outcome^b^	24 (26%)	32 (32%)	0.80 (0.51–1.25)	0.32	0.94 (0.59–1.50)	0.80	47 (51%)	52 (53%)	0.96 (0.73–1.27)	0.78	1.14 (0.85–1.53)	0.38
All-cause mortality	7 (8%)	18 (18%)	0.41 (0.18–0.95)	0.03	–	–	34 (37%)	42 (42%)	0.86 (0.61–1.23)	0.41	1.05 (0.70–1.58)	0.81
Resuscitated cardiac arrest or death	8 (8%)	22 (22%)	0.39 (0.18–0.83)	0.01	–	–	35 (38%)	46 (46%)	0.81 (0.58–1.13)	0.22	0.93 (0.63–1.37)	0.72
Mechanical circulatory support or cardiac transplant	9 (10%)	8 (8%)	1.20 (0.48–2.97)	0.70	–	–	14 (15%)	11 (11%)	1.35 (0.65–2.83)	0.42	1.66 (0.77–3.59)	0.20
Non-fatal myocardial infarction	0 (0%)	0 (0%)	–	–	–	–	0 (0%)	1 (1%)	–	1.00^c^	–	–
Transient ischemic attack or stroke	0 (0%)	0 (0%)	–	–	–	–	0 (0%)	3 (3%)	–	0.25^c^	–	–
Initiation of renal replacement therapy	13 (14%)	11 (11%)	1.26 (0.59–2.67)	0.55	–	–	22 (24%)	15 (15%)	1.56 (0.86–2.82)	0.14	1.73 (0.93–3.20)	0.08

*CI* confidence interval, *RR* relative risk

^a^Adjusted for age, sex, beta-blocker use, inotrope (dobutamine vs. milrinone), and history of atrial fibrillation; relative risk presented is for β(beta-blocker). Due to low event rates in the early resuscitation period, the adjusted relative risk was presented only for the primary composite outcome

^b^Primary composite outcome defined as all-cause mortality, resuscitated cardiac arrest, need for mechanical circulatory support or cardiac transplant, non-fatal myocardial infarction, transient ischemic attack or stroke, or initiation of renal replacement therapy

^c^*P* value for Fisher exact test

### Secondary clinical outcomes

Individual components of the primary composite outcome are summarized in Table 2. With respect to early outcomes, the adjusted relative risk is presented only for the primary composite outcome due to low event rates in the early resuscitation period. In unadjusted analyses, there were fewer early deaths in patients treated with BBs (crude RR (cRR) 0.41; 95% CI 0.18–0.95; *P* = 0.03) (Fig. 2b) and fewer resuscitated cardiac arrests or deaths (cRR 0.39; 95% CI 0.18–0.83; *P* = 0.01) (Fig. 2c). There was no difference in the need for mechanical circulatory support or initiation of RRT. There were no patients who underwent cardiac transplantation, had non-fatal MI, or transient ischemic attack/stroke in the first 48 h of inotropic therapy.

With respect to total in-hospital outcomes, there was no difference in the primary composite outcome (aRR 1.14; 95% CI 0.85–1.53; *P* = 0.38), all-cause mortality, resuscitated cardiac arrest or death, need for mechanical circulatory support, non-fatal MI, transient ischemic attack/stroke, or initiation of RRT in the adjusted models. No patients underwent cardiac transplantation during the index hospitalization.

Secondary safety outcomes are summarized in Table 3. In adjusted analyses, there was no difference in any secondary safety outcome, including CICU length of stay ≥ 7 days, acute kidney injury, arrhythmia requiring intravenous anti-arrhythmic therapy or electrical cardioversion, atrial arrhythmias requiring medical intervention, ventricular arrhythmias, need for initiation or increase of intravenous or oral anti-arrhythmic therapy, and need for initiation or increase of vasopressor therapy.

**Table 3:** Secondary safety outcomes

End point	Beta-blocker (n = 93)	No beta-blocker (n = 99)	Crude RR (95% CI)	*P* value	Adjusted RR^a^ (95% CI)	*P* value
Cardiac intensive care unit length of stay greater than or equal to 7 days	31 (33%)	42 (42%)	0.79 (0.54–1.13)	0.19	0.71 (0.48–1.03)	0.07
Acute kidney injury^b^	86 (96%)	85 (88%)	1.09 (1.00–1.19)	0.051	1.05 (0.92–1.21)	0.47
Arrhythmia requiring medical team intervention^c^	51 (55%)	41 (41%)	1.32 (0.98–1.78)	0.06	1.21 (0.89–1.65)	0.23
Atrial arrhythmia requiring medical team intervention	47 (51%)	32 (32%)	1.56 (1.10–2.22)	0.01	1.33 (0.92–1.91)	0.13
Ventricular arrhythmia^d^	11 (12%)	20 (20%)	0.59 (0.30–1.15)	0.12	0.59 (0.29–1.21)	0.15
Need for oral or intravenous anti-arrhythmic therapy	48 (52%)	36 (36%)	1.42 (1.02–1.97)	0.03	1.30 (0.92–1.85)	0.14
Need for uptitration or addition of vasopressor therapy	91 (98%)	96 (97%)	1.01 (0.96–1.06)	0.70	1.02 (0.86–1.21)	0.84

^a^Adjusted for age, sex, beta-blocker use, inotrope (dobutamine vs. milrinone), and history of atrial fibrillation; relative risk presented is for β(beta-blocker)

^b^Patients with a history of renal replacement therapy prior to randomization were excluded from analysis

^c^Defined as electrical/chemical cardioversion or any intravenous anti-arrhythmia medication administration

^d^Defined as monomorphic or polymorphic ventricular tachycardia greater than 30 s, or hemodynamically unstable ventricular arrhythmia requiring intervention, or ventricular fibrillation

**Fig. 3:**
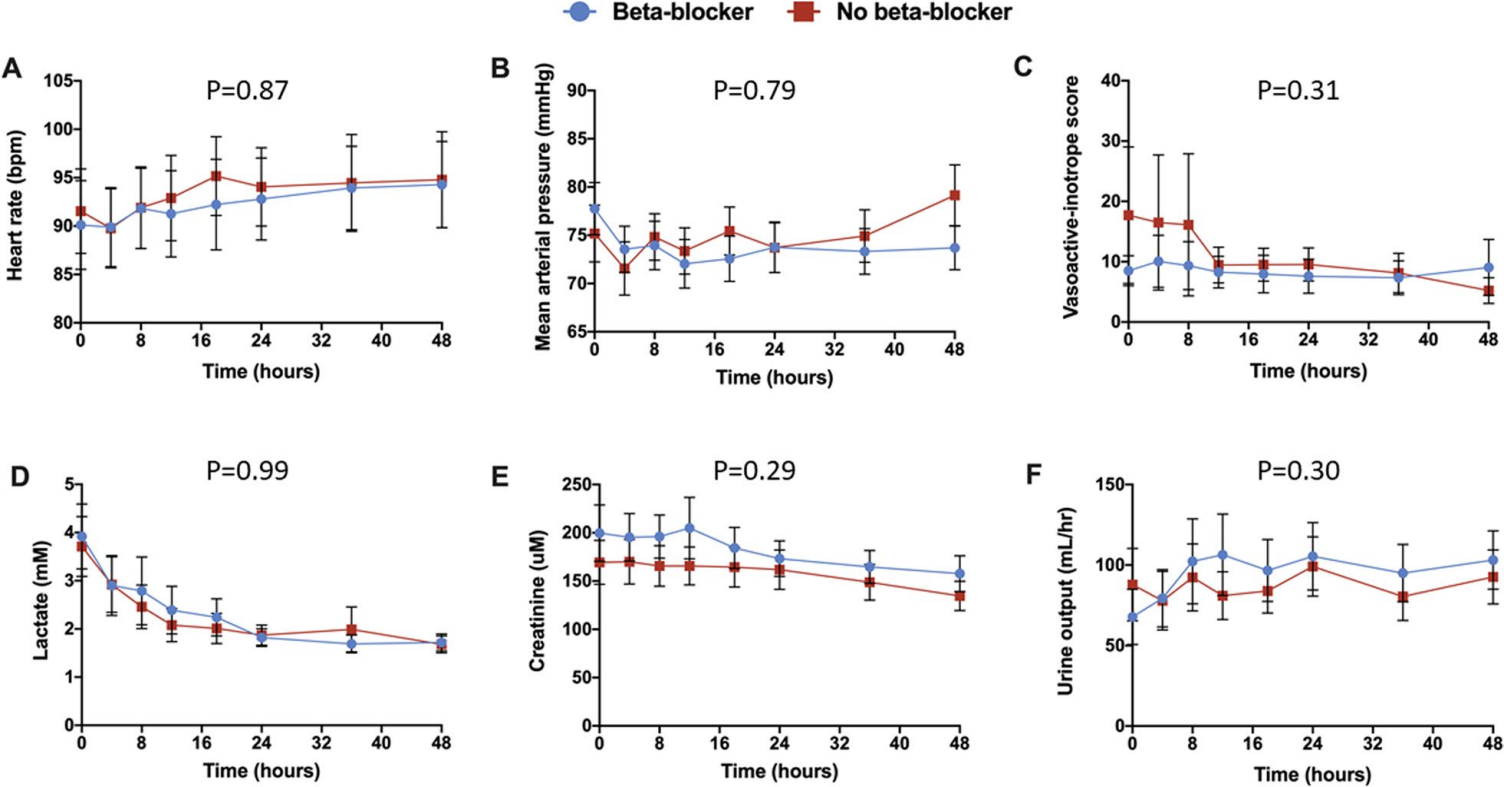
Key clinical and biochemical parameters.** a** Heart rate, **b** mean arterial pressure, **c** vasoactive-inotropic score, **d** serum lactate, **e** serum creatinine, and **f** hourly urine output within the first 48-h of initiation of inotropic therapy. A repeated measure mixed model was used to evaluate differences in continuous variables between groups. All panels demonstrate the mean and 95% confidence intervals between the beta-blocker (blue) and no beta-blocker (red) groups at a specific time interval. *BPM* beats per minute

Overall, no differences were found in heart rate, mean arterial pressure, vasoactive-inotropic score, serum lactate, serum creatinine and hourly urine output between the BB and no BB groups (Fig. 3).

### Impact of BB on hemodynamic parameters in CS

**Fig. 4:**
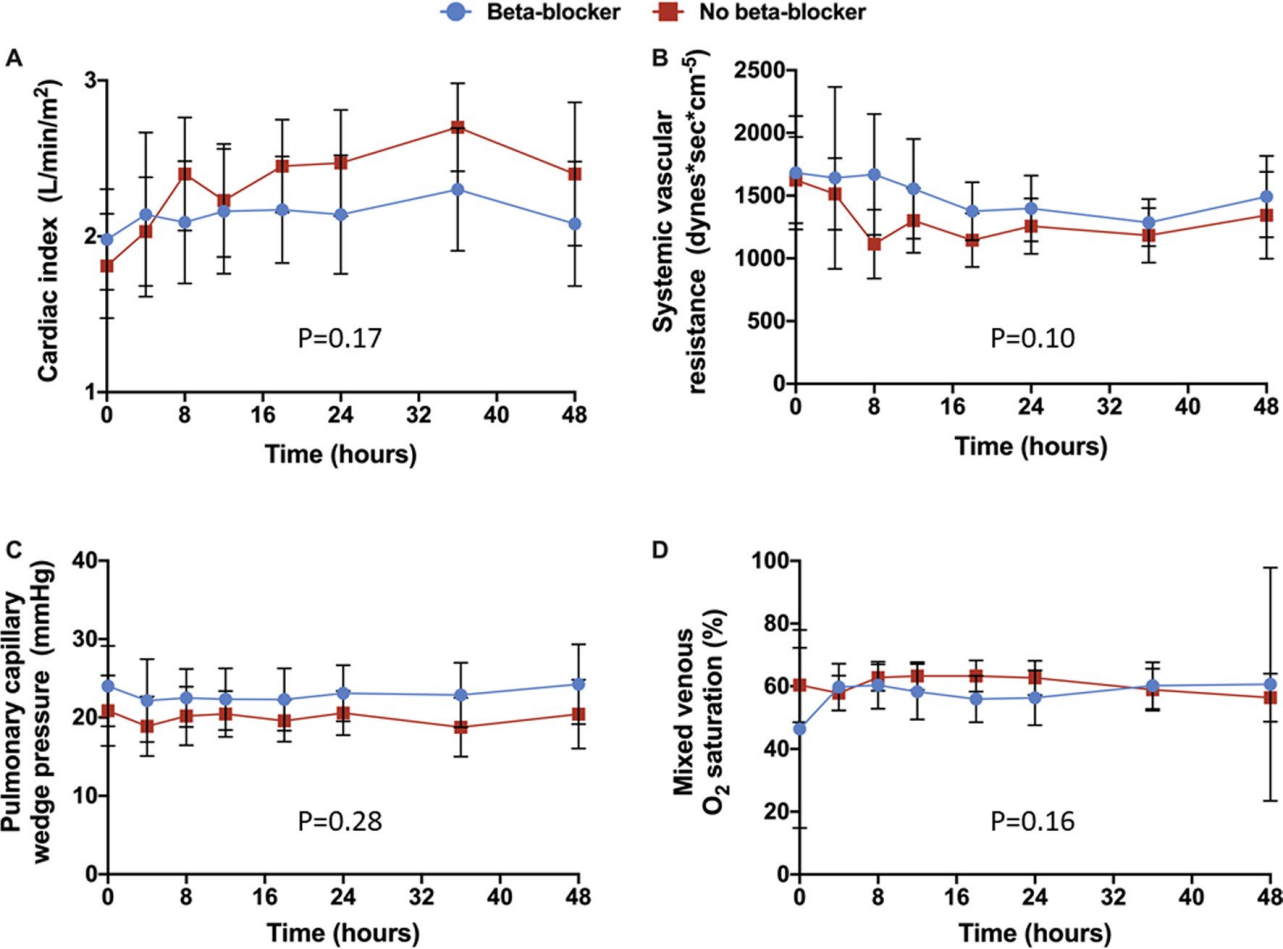
Key hemodynamic parameters.** a** Cardiac index, **b** systemic vascular resistance, **c** pulmonary capillary wedge pressure, and **d** mixed venous oxygen saturation within the first 48-h of initiation of inotropic therapy. A repeated measure mixed model was used to evaluate differences in continuous variables between groups. All panels demonstrate the mean and 95% confidence intervals between the beta-blocker (blue) and no beta-blocker (red) groups at a specific time interval

There were 9 patients in the BB group and 14 patients in the no BB group that had a pulmonary artery catheter at baseline. There were an additional 18 pulmonary artery catheters inserted by the treating teams within the first 48 h following initiation of inotropic therapy. There were no differences in the cardiac index (Fig. 4a), systematic vascular resistance (Fig. 4b), pulmonary capillary wedge pressure (Fig. 4c), or mixed venous oxygen saturation (Fig. 4d) between the two groups.

### Effect of baseline BB use on early response to dobutamine

**Fig. 5:**
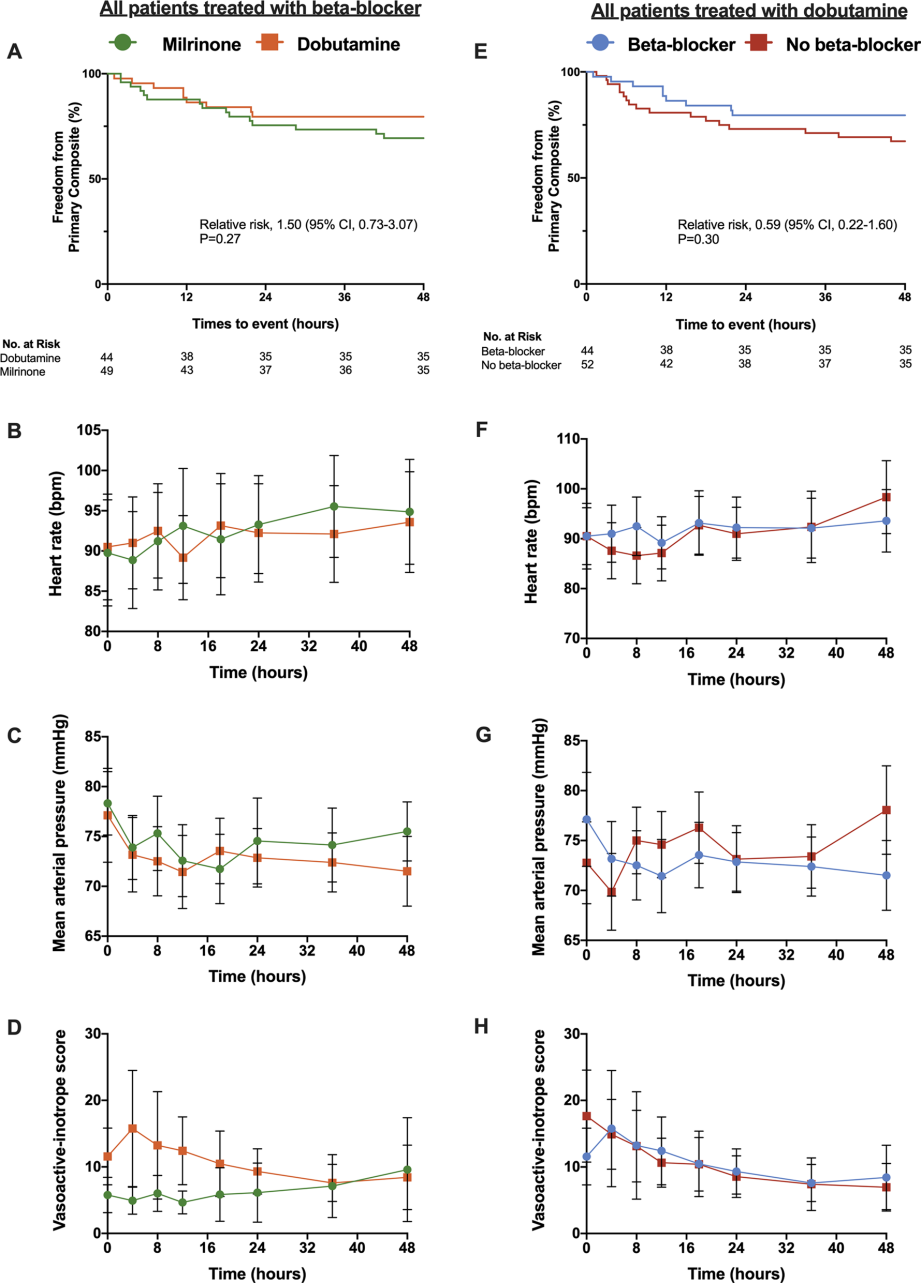
Impact of beta-blocker therapy on dobutamine treated patients. In all patients treated with beta-blockers, there was no difference in **a** primary composite outcome, **b** heart rate or **c** mean arterial pressure when stratified by milrinone (green) and dobutamine (orange). **d** vasoactive inotropic score was different between beta-blocker patients treated with mirlinone versus dobutamine. In all patients treated with dobutamine, there was no difference in **e** primary composite outcome, **f** heart rate, **g** mean arterial pressure, or **h** vasoactive inotropic score when stratified by beta-blocker (blue) and no beta-blocker (red). Survival curves were constructed using Kaplan–Meier estimates. Unadjusted relative risks using Chi-square testing was used for the primary composite outcome. A repeated measure mixed model was used to evaluate differences in continuous variables between groups. **b**–**d** and **e**–**g** demonstrate the mean and 95% confidence intervals between groups. *BPM* beats per minute

There were no differences in the primary composite outcome when evaluating BB-treated patients by dobutamine versus milrinone therapies (cRR 1.50, 95% CI 0.73–3.07; *P* = 0.27) (Fig. 5a). While there were no differences in heart rate (Fig. 5b) or mean arterial pressure (Fig. 5c) between the dobutamine and milrinone BB-treated patients, there was a difference in vasoactive-inotropic score (Fig. 5d; *P* = 0.03).

For patients treated with dobutamine, there was no difference between the BB and no BB groups with respect to the primary composite outcome (Fig. 5e), heart rate (Fig. 5f), mean arterial pressure (Fig. 5g), or vasoactive-inotropic score (Fig. 5h). Lastly, there was no difference in the inotropic titration for patients treated with dobutamine, irrespective of antecedent BB use (*P* = 0.49).

## Discussion

We sought to compare outcomes in patients treated with BBs in the 24 h preceding CS to those who were not on BB therapy. This subgroup analysis of the DOREMI trial provides two important insight for managing patients with CS. First, contrary to our primary hypothesis, BB use was associated with fewer early deaths and cardiac arrests during the resuscitative phase of CS management. However, this protective association was lost during the remainder of the hospitalization period and there was no difference in mortality at hospital discharge. Furthermore, baseline BB use did not appear to influence important hemodynamic parameters and it did not attenuate the effect of dobutamine. In the largest analysis of its type, these findings contradict traditional thinking about the association of baseline BB use in patients with CS.

Contrary to our study hypothesis, BB use was negatively associated with death in the early resuscitation period. As BBs are negative inotropes and chronotropes^14^ and given the need for augmentation of cardiac output in CS, we had expected that there would be worse clinical outcomes in these patients. In addition to their negative inotropic and chronotropic properties, BBs have class II anti-arrhythmic properties by decreasing the spontaneous depolarization of pacemaker myocytes and increasing atrioventricular nodal refractory periods.^14^ After adjustment for clinically important covariates, including a history of atrial fibrillation, there was no difference in atrial arrhythmias requiring intervention but there was a trend toward fewer ventricular arrhythmias in the BB treated group. The observed reduction in early resuscitated cardiac arrest and death, coupled with the trends in reduced ventricular arrhythmias, suggest a paradoxical protective effect of BBs early in the resuscitative period when vasoactive and adrenergic stimulation are maximal. The role for BB therapy to modulate arrhythmia risk early in CS warrants consideration and further study.

In the DOREMI trial, patients with CS were randomly assigned to receive dobutamine or milrinone, the two approved inotropic agents in North America. Milrinone is a phosphodiesterase-III inhibitor and dobutamine is a synthetic catecholamine with beta-1 and beta-2 receptor agonism. In patients with acute decompensated heart failure, the inotropic effects of dobutamine have been shown to be dependent on the degree of occupancy of the beta-receptor and on the activity of the beta-adrenergic signal transduction mechanisms.^11,15^ As a result, the use of a phosphodiesterase-III inhibitor has been suggested in lieu of beta-agonists for patients who had been treated with BBs.^16^ Our study is the first to evaluate the impact of baseline BB on clinical and hemodynamic parameters and contrary to traditional teaching, we found little difference. While vasoactive-inotrope score was mildly greater among BB patients treated with dobutamine compared to milrinone, there was no difference in heart rate, mean arterial pressure or clinical outcomes. Moreover, there was no difference between BB and non-BB vasoactive-inotrope scores in those randomized to dobutamine. Contrasting with patients with decompensated heart failure, in the shock state with hypotension there is increased sympathetic tone and catecholamine levels in a compensatory response to maintain cardiac output,^17^ which may mitigate the BB effect. Accordingly, selection of inotropic agent could reasonably be based on physician comfort and hemodynamic goals of the patient irrespective of baseline BB use.

### Limitations

Our study is not without limitations. First, this is a subgroup analysis of the DOREMI study which compared dobutamine and milrinone in CS. While the trial did not stratify by BB usage, nearly half of the participants in the trial were on treatment with BBs in the 24 h prior to randomization. This provided us with a high-quality dataset of CS patients with and without BB use in which the potential for selection bias in inotrope selection by the treating physicians was minimized. Second, BBs have varying receptor selectivity and vasodilating properties. In this study, we report the impact of BBs as a class effect and did not stratify by BB type; however, nearly all patients were treated with one of three commonly used cardioselective BBs (i.e. metoprolol, bisoprolol, or carvedilol). Lastly, pulmonary artery catheter use was selective, limiting hemodynamic response to a quarter of study participants. Nonetheless, heart rate, blood pressure response and vasoactive-inotropic scores were available for all patients and showed no differences among the BB and non-BB groups.

## Conclusion

In patients with CS who were treated with BBs in the 24 h prior to initiating inotropic therapy, there were fewer deaths and resuscitated cardiac arrests in the early resuscitation period, although this difference was no longer present by hospital discharge. BB therapy was not associated with an impaired hemodynamic response to inotropic therapy in CS, including with dobutamine. Modulating arrhythmic risk in CS may offer a mechanism to reduce adverse outcomes in CS.

## Data Availability

The datasets used and/or analysed during the current study are available from the corresponding author on reasonable request.

## Abbreviations

BB: Beta-blocker
CICU: Cardiac intensive care unit
CS: Cardiogenic shock
MI: Myocardial infarction
RRT: Renal replacement therapy
